# Evaluating responses to temperature during pre-metamorphosis and carry-over effects at post-metamorphosis in the wood tiger moth (*Arctia plantaginis*)

**DOI:** 10.1098/rstb.2019.0295

**Published:** 2019-08-26

**Authors:** Juan A. Galarza, Kishor Dhaygude, Behnaz Ghaedi, Kaisa Suisto, Janne Valkonen, Johanna Mappes

**Affiliations:** 1 Department of Biological and Environmental Sciences, University of Jyväskylä, 40014 Jyväskylä, Finland; 2 University of Helsinki, 00100 Helsinki, Finland

**Keywords:** life stage autonomy, wood tiger moth, transcriptome, carry-over effects, melanization

## Abstract

Insect metamorphosis is one of the most recognized processes delimiting transitions between phenotypes. It has been traditionally postulated as an adaptive process decoupling traits between life stages, allowing evolutionary independence of pre- and post-metamorphic phenotypes. However, the degree of autonomy between these life stages varies depending on the species and has not been studied in detail over multiple traits simultaneously. Here, we reared full-sib larvae of the warningly coloured wood tiger moth (*Arctia plantaginis*) in different temperatures and examined their responses for phenotypic (melanization change, number of moults), gene expression (RNA-seq and qPCR of candidate genes for melanization and flight performance) and life-histories traits (pupal weight, and larval and pupal ages). In the emerging adults, we examined their phenotypes (melanization and size) and compared them at three condition proxies: heat absorption (ability to engage flight), flight metabolism (ability to sustain flight) and overall flight performance. We found that some larval responses, as evidenced by gene expression and change in melanization, did not have an effect on the adult (i.e. size and wing melanization), whereas other adult traits such as heat absorption, body melanization and flight performance were found to be impacted by rearing temperature. Adults reared at high temperature showed higher resting metabolic rate, lower body melanization, faster heating rate, lower body temperature at take-off and inferior flight performance than cold-reared adults. Thus, our results did not unambiguously support the environment-matching hypothesis. Our results illustrate the importance of assessing multiple traits across life stages as these may only be partly decoupled by metamorphosis.

This article is part of the theme issue ‘The evolution of complete metamorphosis'.

## 1. Introduction

Most insects exhibit different phenotypes across their lifespan that differ radically in form and function [1]. Different phenotypes usually experience different environments, and hence, are subject to different selection pressures. The differences in selective environments are likely to cause a shift in the selective value of traits across ontogeny [2]. For instance, maximizing fitness during a particular phenotype could alter the conditions for performance and selection in a later phenotype. Therefore, to fully understand how selection shapes phenotypic variation, we must first understand the degree of autonomy between life stages.

Insect metamorphosis is one of the most recognized processes delimiting transitions between phenotypes. It has been traditionally postulated as an adaptive process decoupling traits between life stages, allowing evolutionary independence of pre- and post-metamorphic phenotypes [2,3]. Numerous techniques are currently available for metamorphosis visualization [4]. In holometabolous insects, larvae undergo several moults (or ecdysis) before metamorphosis in which the old cuticle is shed and a new one is produced allowing the insect to grow [5]. It has been shown that moulting is a period of high activity and regulation of several processes such as digestion, transport, proteolysis and cell death [6–9]. Likewise, larval moults can vary in number and frequency in response to environmental conditions such as temperature and diet [10,11]. Thus, the larval stage is highly responsive at many functional levels. To what extent larval responses to developmental conditions can influence metamorphosis, and thereby impact the fitness of the subsequent adult phenotype, is poorly understood.

In insects, a few studies have empirically tested the coupling of the pre- and post-metamorphic phenotypes and have found varying results, suggesting that life stages are not fully independent, and that some effects can be carried over across the metamorphic boundary [12–15]. For instance, photoperiod and nutritional manipulation during the larval stage of the damselfly *Lestes viridis* did not show significant effects on adult size [12]. On the other hand, larval exposure to UV in the damselfly *Coenagrion puella* translated into smaller adult size with lower melanotic encapsulation response [14]. Congruently, heat stress on eggs of the butterfly *Bicyclus anynana* showed negative fitness effects carried over to the larvae and adults as evidenced by decreased survival, growth and body mass [16]. In the winter moth (*Operophtera brumata*), phenological shifts induced by photoperiod manipulation during the larval stage were partly compensated in the subsequent life stages [17]. Most studies, however, have evaluated carry-over effects (or their lack of) for a single or few quantitative traits at a time (i.e. size, fecundity), ignoring their molecular bases or associated physiological changes.

Here, we take an integrative approach measuring a suite of traits relating to different functions (warning coloration and flight performance) at different levels (gene expression to organismal) of pre- and post-metamorphic wood tiger moth (*Arctia plantaginis*) exposed to two temperature conditions during larval development. The wood tiger moth displays a warning coloration against potential predators both in the larval and adult phenotypes that can easily be tracked [18,19], and its ecological function is well established [20,21].

Three possible outcome scenarios could be expected. (A) A continuous response to the thermal environment across instars, that translates into better condition of the adult phenotype. (B) Larval responses but no condition gains for the adults. (C) No responses across instars and no condition gains for adults. Scenario A would imply that larval responses are carried-over across life stages, whereas in scenario B metamorphosis effectively decouples life stages. In turn, scenario C would indicate that trait decoupling occurs across larval instars rather than in the more dramatic larva–adult transition. We also tested if the thermal environment that the larvae are exposed to makes adults perform better in the same environment (so-called environment-matching hypothesis). We used a combination of approaches in trying to disentangle these scenarios and their underlying mechanisms.

## 2. Material and methods

### (a) Study species

The wood tiger moth is an aposematic species widely distributed throughout the Holarctic [22]. Larvae display a red patch against an otherwise dark body. The patch is variable in size and functions as a warning signal against avian predators [20]. In Finland, the larval phase can last for almost 1 year starting from approximately the end of June-mid-July, until approximately mid-May-early June, when after 7–14 days of pupation adults emerge. The mating season lasts approximately two to three weeks and the egg stage lasts 5–10 days in the laboratory. In the laboratory, diapause can be broken down and it is possible to grow several generations within the year.

### (b) Sample collection

Samples were produced using a split-family rearing design including seven families (F1–F7) from the laboratory stock at the University of Jyväskylä, Finland. A total of 60 full-sib larvae per family were reared in two different temperature environments; high (H), 25°C (*n* = 30/family) and low (L), 16°C (*n* = 30/family). Both temperatures are within the range that wild larvae experience in Finland during development around July. Our goal was to create two different thermal environments in which temperature remained constantly high and constantly low relative to each other. The temperature inside the growing chambers (Sanyo MLR-351) remained constant with a 12 h light/dark cycle for both temperature treatments and the larvae were randomized within the chambers every other day. Ambient temperature was chosen as the variable factor to exclude confounding effects of heat gain/losses caused by light radiation. While ectotherms benefit greatly from light radiation for heat absorption, they must first actively seek light radiation starting from the ambient temperature. Thus, the ability to perform at ambient temperature reflects a more basal thermal sensitivity. Larvae were fed wild dandelion (*Taraxacum* spp*.*) collected every second day in the vicinity of the University of Jyväskylä. Newly hatched larvae show a homogeneous greyish coloration until the third instar, when body segments turn black or red to display the warning coloration. At this point, larvae were placed individually in Petri dishes and the number of black body segments recorded.

Larvae were monitored for moults every second day. Larval moults are easily detected by the presence of shredded skin inside the Petri dish. When a moult had occurred, the number of black body segments was compared to the previous instar for increases (I), decreases (D) or no change (N) in the number of black segments. For instance, a moulting that resulted in gains of 2 black body segments was scored as +2. In the same way, moults that reduced the number of black body segments were scored negatively (i.e. −2), whereas moults that did not have an effect on the number of black body segments were scored as 0. Rearing continued until all larvae had gone to pupation or died. The emerging adults from both treatments were maintained in their respective temperature environment between 32 and 40 h after eclosion. According to our previous experiments, newly eclosed adults reach sexual maturity within 25 h of eclosion [23].

### (c) Transcriptome analyses

We selected larvae that had undergone one or multiple moults for RNA sequencing. For instance, larvae that had moulted one time, two times and up to four times, were placed in RNAlater stabilizing solution (Qiagen, Valencia, CA, USA) immediately after the moult was detected. We consider each moult as a transition to a new instar. Hence, instars are denoted as 1–4 henceforth. For RNA-Seq, we sequenced two families (F3, F7) and, whenever possible, we sequenced more than one larva/instar/family/treatment as biological replicates (table 1). All samples were kept at −20°C until RNA extraction. Total RNA was extracted using RNeasy Mini Kit (Qiagen) according to the manufacturer's instructions with additional TriReagent (MRC, Inc.) and DNase (Qiagen, Valencia, CA, USA) treatments. The quality and quantity of total RNA were inspected in a BioAnalyzer 2100 using RNA 6000 Nano Kit (Agilent). Subsequently, mRNA was isolated by means of two isolation cycles using Dynabeads mRNA purification kit (Ambion^®^) and quantified using RNA 6000 Pico Kit in a BioAnalyzer 2100 (Agilent). Pair-end (2 × 100 pb) cDNA libraries were constructed for each sample according to Illumina's TruSeq Stranded HT protocol. The libraries were individually indexed and sequenced in an Illumina HiScanSQ sequencer at the DNA sequencing and genomics laboratory, Institute of Biotechnology of the University of Helsinki, Finland.

**Table 1.** Number of samples by treatment, family and instar from both RNA-seq and qPCR datasets.

	number of different families		no. samples/treatment qPCR		no. samples/treatment RNA-seq	
instar	qPCR	RNA-seq	H	L	H	L
1	6	2	9	7	4	3
2	5	2	6	7	5	5
3	6	2	6	8	3	4
4	2	2	2	7	2	4

### (d) Reads processing and mapping

The quality of the raw reads from the libraries was first inspected with FastQC (http://www.bioinformatics.babraham.ac.uk/pro-jects/fastqc/) and summarized using MultiQC v. 0.8 [24]. Based on this initial quality check, we used the FASTX toolkit (http://hannonlab.cshl.edu/fastx_toolkit/) to remove low-quality bases and sequencing artefacts. Bases with a Phred quality score of less than 25 were filtered out, and reads shorter than 85 bases after trimming were removed. Pair-end reads were then sorted and synchronized using custom bash scripts.

To calculate expression profiles of the different samples, we aligned the high-quality reads to the wood tiger moth's reference transcriptome [25]. Briefly, we first indexed the reference transcriptome and aligned the reads using bowtie2 v. 2.2.5 [26]. The alignments were then converted to binary format and the number of mapped reads for each sample counted using SAMtools v. 1.3.1 [27] and the read counts were merged into a single read count table for downstream expression analyses. To evaluate expression profiles of the candidate genes, we first obtained a normalized expression by dividing the number of reads that mapped to each candidate gene transcript (Mr) by the total number of reads (Tr), multiplied by the transcript length (Tl) scaled by a factor of a million (i.e. (Mr/Tr) × Tl^10^9^). This procedure returns normalized counts as transcripts per million reads sequenced (TPM), in which the sum of all TPMs in each sample are the same, thus allowing a direct comparison of normalized expression values across samples and treatments.

### (e) Gene expression and annotation

To obtain an overview of which and how many genes are impacted, we performed differential expression analyses on the RNA-seq data using the R package edgeR [28]. Gene expression was tested under a quantile-adjusted conditional maximum-likelihood (qCML) framework setting individual contrast within instars between the treatments (i.e. Instar1 H versus Instar1 L) applying the *exactTest* function. Subsequently, we obtained a functional annotation of the identified upregulated and downregulated genes by blasting (BLASTx) [29] against a non-redundant protein database (nr) (NCBI; last updated 30 November 2018). After blasting, all hits that showed less than 70% amino acid identity, sequence length of less than 200 bp and e-value ≤ 10^−5^ were filtered out. Gene ontology terms (GO) and information of the protein family was obtained using Blast2Go v. 4 [30].

### (f) Candidate genes and their expression during development

We aimed to evaluate the link between variation in melanization (i.e. plasticity) during the larval phenotype as well as its functional significance for the reproductive adult phenotype. We followed the molecular mechanism behind the observed plasticity by examining four core melanin synthesis genes across larva-to-larva moults by means of quantitative polymerase chain reaction (qPCR). This qPCR dataset includes all the samples of both treatments from the rest of the families that were not RNA-sequenced (table 1). The genes examined (*DOPA decarboxylase* (*Ddc*), *yellow*, tyrosine hydroxylase (*Th*) and *laccase2*) have been reported to impact melanization in Lepidoptera [31–33]. We also evaluated whether the expression of these genes changed when the number of black body segments had increased (I), decreased (D) or remained the same (N) after the immediate previous larval moult. The candidate gene mRNA sequences from other Lepidopterans were obtained from the National Centre for Biotechnology Information (NCBI). The sequences were then searched for orthology against the wood tiger moth's reference transcriptome [25] through their protein translation to all six possible frames using tBLASTx. Transcripts with greater than 85% sequence similarity and greater than 300 bp alignment length were selected and blasted (BLASTx) back to NCBI to confirm orthology. In the same manner, we selected four candidate genes (myofilin (*Mf*), flightin (*Fln*), triosephosphate isomerase (*tpi*) and phosphoglucose isomerase (*pgi*)), known to impact flight performance in Lepidopterans [34–37]. As with the melanin genes, we aimed at relating the expression of these genes during the larval phase with adult flight performance (see below). The orthologue transcript sequence and accession numbers of the candidate genes are given in electronic supplementary material, table T1S.

We mapped the orthologue transcripts of each gene to an *A. plantaginis* draft genome assembly (J. A. Galarza, C. W. Wheat, J. Mappes 2015, unpublished) to identify exon-intron boundaries using Mummer v. 3.23. Bridging primers for qPCR were designed using Primer3 v. 4.0.0 [38]. As normalization controls (i.e. housekeeping genes), we selected two transcripts from the RNA-seq data which showed uniform expression within and between the two temperature treatments. The software Normfinder v. 5 [39] was used to evaluate the normalized counts matrix to find the transcripts with the highest stability value and lowest expression variation within and between the two temperature treatments. The primer sequences are given in electronic supplementary material, table T1S.

Total RNA for qPCR was extracted and purified as described above from larvae of both temperature treatments. High-quality RNA (200 ng) was converted into cDNA using the iScript cDNA synthesis kit (Bio-Rad). The specificity, dynamic range and PCR efficiency of each primer pair was determined by testing against a six-step twofold dilution series of cDNA. All genes were amplified in triplicate (i.e. as technical replicates) in at least two biological replicates (i.e. within each instar and treatment; table 1). An inter-run calibrator was prepared by pooling cDNA from all samples from both temperature treatments and included in all PCRs runs. All PCRs (20 µl final volume) were run on a CFX96 (Bio-Rad™) thermocycler using 300 nM of each primer, 10 µl iQ SYBR® Green Supermix (Bio-Rad™) and 4 µl of cDNA diluted 20-fold. PCR conditions used throughout were 95°C for 3 min followed by 40 cycles of 95°C for 10 s, 60°C for 15 s and 72°C for 10 s. Melt curves were run after amplification to check for specificity from 55°C to 95°C with fluorescence readings taken in 0.5°C increments. Amplification efficiency of each gene was calculated by plotting the standard curve *Cq* values against the log of the dilution factor for each point on the curve. The relative gene expression between the treatments was examined following the methods of Vandesompele *et al.* [40] developed for using multiple housekeeping genes,

2.1 $$\mathrm{relative}\;\;\mathrm{GE}=\;\frac{(E_{\mathrm{GOI}}){\;}^{\Delta Ct\;\mathrm{GOI}}}{\mathrm{Geom}\;\mathrm{Mean}\;[(E_{\mathrm{REF}}){\;}^{\Delta Ct\;\mathrm{REF}}]},$$

where *E* refers to the primer efficiency, *Ct* the PCR threshold cycle of the gene of interest (GOI) in the nominator, divided by the geometric mean of all relative quantities (*E*_REF_)*^Δ^*^CtREF^ of the housekeeping genes (REF).

Because the number of RNA-seq libraries was not the same for each larva/instar/family/treatment (table 1), we implemented a 2-ANOVA type iii for imbalance designs using the R package Car [41] to test if the treatment, family or their interaction had an effect on the TPM counts with the following model: *Anova(lm(TPM* ∼ *Treatment* *+* *Family* *+* *Treatment:Family* *+* *Instar, contrasts* *=* *list(Treatment* *=* *contr.sum, Instar* *=* *contr.sum, Family* *=* *contr.sum, Instar* *=* *contr.sum)), type* *=* *3).* No significant effects were observed except for the treatment (*p* *<* 0.0001). We thus performed all-versus-all pairwise *t*-tests of the TPM counts to identify expression differences within and between instars and treatments. All comparisons were performed in R (Core Team 2017) correcting for multiple testing by the false discovery rate method using the *pairwise.t.test* function.

### (g) Life-histories: developing phenotype

We evaluated several life-history traits of the developing phenotypes (larvae + pupae) from both thermal environments. For the larvae, we examined the number of moults before pupation, as well as the time until pupation (i.e. larval age). The total number of moults was recorded as the sum of all increase, decrease and neutral moults observed. Larval age was recorded as the number of days spent in the larval stage since the larvae were first placed into the two thermal environments until their pupation. For the pupae, we examined their weight and the time until their eclosion (i.e. pupal age). Pupal weight was recorded in milligrams using a Mettler Xs204 digital scale and rounded up to the nearest hundredth. Pupal age refers to the number of days spent at the pupal stage including the day of pupation and day of hatching. We used the R package lme4 v. 1.1-15 [42] to test if the treatment impacted the number of moults and larval age, setting the family as a random effect in the following model: *lmer(Y* ∼ *Treatment* *+* *(1|Family))*. For the pupal weight and pupal age, we included the sex in the model as it can already be distinguished during pupation: *lmer(Y* ∼ *Treatment* *+* *Sex* *+* *Treatment:Sex* *+* *(1| Family))*.

### (h) Adult image analyses

A total of 25 adult females (H = 7, L = 18) and 52 adult males (H = 18, L = 34) were examined using digital image analyses for their proportion of melanization in the abdomen, as well as in the fore- and hindwings. We also measured the area of the bodies (thorax + abdomen), and the area of both wing sets. Frozen adults were placed in UV-sterilized airtight jars for relaxation with cotton moistened with 90% ddH2O-10% antiseptic solution to avoid mouldering for 24–36 h. After relaxation, the adults were pinned to wooden mounting blocks with their wings fully spread for 48 h. Wings were then separated from the bodies and photographed together using a FujiFilm, FinePix S3Pro digital camera setting an exposure time of 1/30 and ISO = 160. To analyse the proportion of melanization, we selected the regions of interest (i.e. black areas in bodies or wings) from the digital images using ImageJ v. 1.46r [43] (electronic supplementary material, figure F1S). The areas of wings and bodies were measured by selecting these regions and converting the number of pixels within these regions to square centimetres using ImageJ v. 1.46r [43]. The proportion of melanization was obtained by dividing the total area by the melanized parts. A mixed effect model *lmer(Y* ∼ *Treatment* *+* *Sex* *+* *Treatment:Sex* *+* *(1| Family))* was implemented for the analyses of these regions of interest.

### (i) Adult condition: heating assay

A total of 20 adult females (H = 7, L = 13) and 44 males (H = 24, L = 20) were tested for their heat absorption capacity and the body temperature needed to engage flight using infrared thermography. In this assay, we aimed to determine if the temperature experienced during development could impact these traits in adults. First, a 2.5 cm thread was glued to the dorsal side of the thorax (electronic supplementary material, figure F2S) and the moth's initial body temperature was measured with an infrared digital camera (FLIR Systems AB, colour profile sRGB IEC61966-2.1, exposure time 1/250) and immediately placed in a thermal chamber (a modified thermoblock) hanging from the thread (electronic supplementary material, figure F2S). The temperature inside the thermal chamber was set to ±0.5°C of the moth's initial temperature and increased at the rate of 1°C min^−1^ until the moth engaged in active flight. At this point, its body temperature was measured with the infrared camera and the time elapsed recorded (i.e. take-off time). We then calculated the heating rate as the difference between the final and initial body temperature divided by the time to take-off. Differences between samples from both thermal environments were tested within the sexes using the following model (*lmer(Y* ∼ *Treatment + Sex + Treatment:Sex + (1|Family))*.

### (j) Adult condition: metabolic assay

As another condition proxy, we measured the metabolic rate from the same adults of the heating assay. Here, we performed a reciprocal assay in which adults were evaluated for their capacity to fly in the temperature in which they developed as larvae, as well as in the other temperature treatment. Here, we aimed to determine if the temperature experienced as larvae could impact the adult metabolic rate when flying at different temperatures. The assays were conducted using the same metabolic chamber (CO_2_ analyzer, LI-6252; LI-COR, Lincoln, NE, USA) in two rooms at different temperatures (16°C and 25°C), with a 24-h difference between assays. Prior to the assay, the adults were placed at the opposite treatment's temperature for 12 h as an acclimation period. The chamber was calibrated with CO_2_-free air and span gas. The same gas bottle (CO_2_ 300 ppm AGA, Finland) was used in all assays setting a constant 100 ml min^−1^ flow. In each assay, a moth was placed inside the metabolic chamber for 7 min to get accustomed to the chamber. After this period, the moth was left undisturbed for five minutes and its metabolic rate recorded. This was considered as the basal or resting metabolic rate. Then, the moth was forced to fly by manually shaking the chamber for five minutes and its peak metabolic rate was recorded. During this step, we evaluated the flight performance measured as the time at flight divided by the number of times the moth stopped flying. For instance, a rank of 5 was given if the moth did not stop flying during the 5-min flight trial. We then left the moth undisturbed and recorded the time elapsed until it reattained its resting metabolic rate (i.e. recovery rate) (electronic supplementary material, figure F3S). A shorter recovery rate was considered an indicator of better condition [44]. For analyses, the metabolic measurements were corrected by the individual's weight measured when a pupa, and the individual ID was included in the mixed-effect models to account for repeated measures given that the same individuals were tested in both temperature treatments. We implemented a backward reduced modelling approach starting with the most complex model *lmer(Y* ∼ *Treat.Origin* *+* *Treat.Exp* *+* *Treat.Origin:Treat.Exp* *+* *Sex* *+* *Treat.Origin:Sex* *+* *Treat.Exp:Sex* *+* *(1|ID)* *+* *(1|Family))* dropping one term at the time using the *step* function in the lmerTest v. 3.1 R package [45]. The final model chosen was *lmer(Y* ∼ *Treat.Origin* *+* *Treat.Exp* *+* *Sex* *+* *(1|ID))*.

## 3. Results

Here, we reared full-sib larvae from seven families in two different temperature environments—high (H), 25°C (*n* = 30/family) and low (L), 16°C (*n* = 30/family)—and examined their responses by phenotypic (melanization change, number of moults), gene expression (RNA-seq and qPCR of candidate genes for melanization and flight performance) and life-histories traits (pupal weight, and larval and pupal ages). From the emerging adults, we examined their phenotypes (melanization and size) and compared them at three condition proxies: heat absorption (ability to engage flight), flight metabolism (ability to sustain flight) and overall flight performance.

### (a) Larval moults

**Figure 1.**
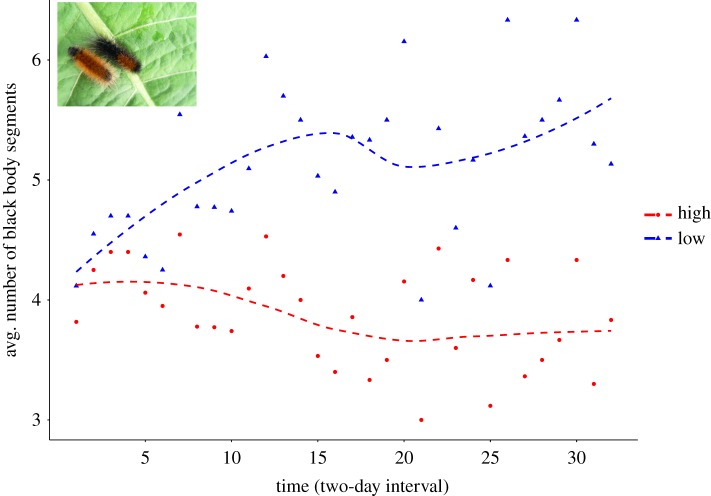
Average number of black body segments in developing larvae of the wood tiger moth (*Arctia plantaginis*) in high (H) and low (L) temperature. (Online version in colour.)

Larvae from the L treatment gradually increased the number of black body segments. The opposite was observed in their full-sibs in the H treatment, which increased their red warning coloration (figure 1). This was consistent across all families (electronic supplementary material, figure F4S). Likewise, larvae from the L treatment underwent more moults than their full-sibs in the H treatment (electronic supplementary material, figure 8S).

### (b) Reads processing and mapping

After quality filtering and trimming, a mean of ≈22 million reads per sample were obtained. The number of reads/sample/treatment/family is presented in electronic supplementary material, table T2S. These high-quality reads were used for downstream analyses of gene expression.

### (c) Gene expression and annotation

We identified a total of 1664 differentially expressed genes (DEG) from both families between the H and L treatments. We found significantly expressed genes within all instars between the treatments with a higher number of differences between later instars (table 2). Most of the DEG were downregulated in larvae from the L treatment according to our contrasts results. The biological processes and molecular functions that the upregulated and downregulated genes are putatively involved in are shown in electronic supplementary material, figure F5S, and their full annotation is presented in electronic supplementary material, tables T3S–T12S. We observed different processes taking place between L and H treatments. Processes involved in growth such as structural constituents of cuticle and chitin binding were mostly downregulated in larvae from the L treatment, whereas oxidation–reduction and innate immune responses were upregulated in larvae from the H treatment (electronic supplementary material, figure F5S).

**Table 2.** Number of upregulated and downregulated differentially expressed genes (DEG) within instars between high (H) and low (L) temperature treatments in wood tiger moth (*Arctia plantaginis*) larvae.

comparison	total DEG	upregulated in H versus L	downregulated in L versus H
Inst1 H versus Inst1 L	375	119	256
Inst2 H versus Inst2 L	395	110	285
Inst3 H versus Inst3 L	646	199	447
Inst4 H versus Inst4 L	640	383	257

### (d) Candidate genes

**Figure 2.**
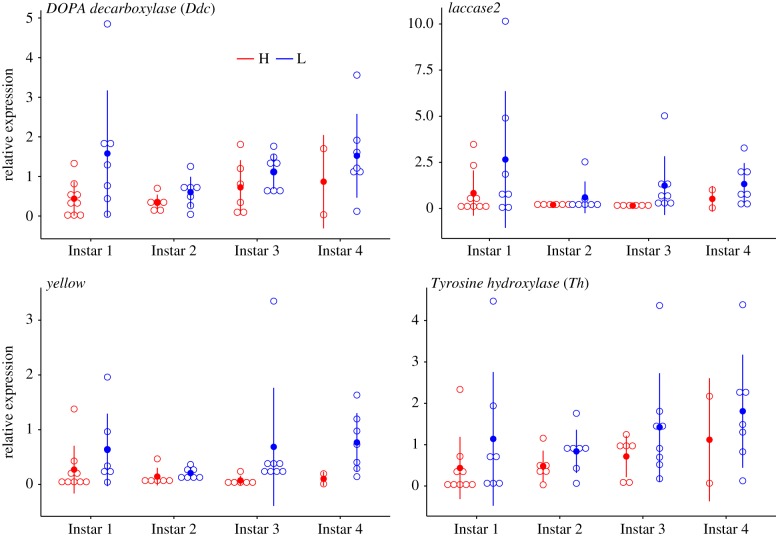
Expression patterns of melanin candidate genes in wood tiger moth (*Arctia plantaginis*) larvae reared at high (H) and low (L) temperature. (Online version in colour.)

**Figure 3.**
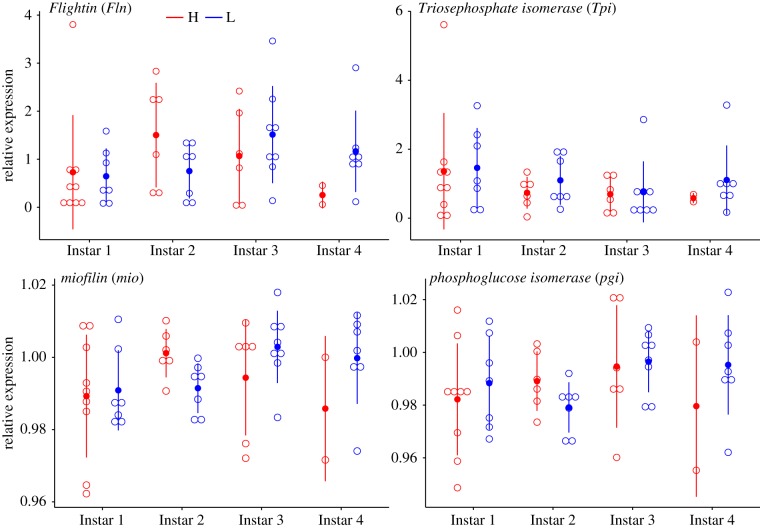
Expression patterns of flight performance candidate genes in wood tiger moth (*Arctia plantaginis*) larvae reared in high (H, red) and low (L, blue) temperature treatments. (Online version in colour.)

**Figure 4.**
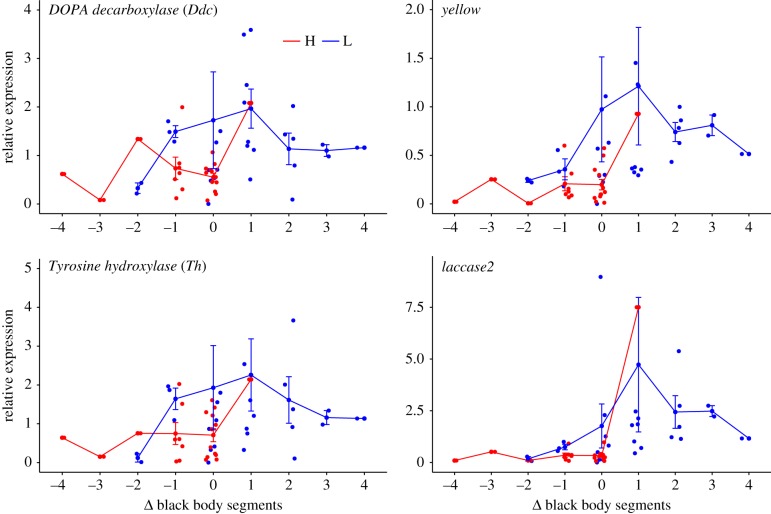
Expression patterns (*y*-axis) of melanin candidate genes in wood tiger moth (*Arctia plantaginis*) larvae after a moult in which the number of black body segments (*x*-axis) increased (positive numbers), decreased (negative numbers) or remained the same (zero) after the previous moult. High (H, red) and low (L, blue) temperature treatments. (Online version in colour.)

The majority of significant pairwise gene expression differences as inferred from the RNA-seq data between instars and treatments occurred at instar 1 from the H treatment, which differed from the other instars and their interaction with treatments (table 3; electronic supplementary material, table T13S). This suggests an immediate response of the larvae when placed in high temperature. Likewise, as evidenced from the qPCR data, the majority of candidate genes showed differentiation in their relative expression during the latest instar between H and L treatments (figures 2 and 3). Across instars, however, there was an overall greater expression in melanin genes in the L treatment, except at the last instar, whereas no clear pattern was observed in the flight performance genes (figures 2 and 3; electronic supplementary material, table T13S). The majority of the moults were neutral (i.e. neither decrease or increase of black body segments) (figure 4). Within treatments there was a trend of lower expression when larvae had decreased black body segments, being overall lower in larvae from treatment H. Significant differences, however, were observed between the treatments in all genes for decreases in black segments as inferred from the RNA-seq dataset (electronic supplementary material, table T13S). There were no differences between treatments for increased or neutral moults (figure 4; electronic supplementary material, table T13S).

**Table 3.** Summary of significant (*p* < 0.05) pairwise gene expression differences in candidate melanin and flight performance genes. Only significant comparisons are indicated by gene. d, *DOPA decarboxylase*; y, *yellow*; l, *laccase*; T, *tyrosine hydroxylase*; m, miofilin; f, flightin; t, triosephosphate isomerase; p, phosphoglucose isomerase; H , high temperature treatment; L, low temperature treatment. Bold font indicates melanin genes, italic font indicates flight performance genes.

gene	Instar1 H	Instar1 L	Instar2 H	Instar2 L	Instar3 H	Instar3 L	Instar4 H	Instar4 L
Instar1 L	**d,y**,*m*	—	—	—	—	—	—	—
Instar2 H	**d**		—	—	—	—	—	—
Instar2 L		**T**	**l,T,y**		—	—	—	—
Instar3 H	**y**,*t*		**y***,t*		—	—	—	—
Instar3 L		*m*		*m*	*p*	—	—	—
Instar4 H	**d**,*m*		*m*		**d,T,l***,f,t,p*	—	—	*m,t,p*
Instar4 L	**d**,*t*	*t*				*m*	*m,t*	—

### (e) Life-histories: developing phenotype

Our results showed that larvae from all families reared in the L treatment underwent almost twice the number of moults than their siblings in the H treatment (electronic supplementary material, figure F8S). Likewise, larval and pupal ages were both older in the L treatment, whereas the females from the L treatment showed a lighter weight when pupae (electronic supplementary material, figure F9S). All differences were statistically significant (*p* *<* 0.001) for the treatment effect in our mixed-effect models, and only one significant interaction (*p* *<* 0.001) was detected between the treatment and the sex when analysing pupal weight (electronic supplementary material, tables T14S–T17S).

### (f) Adult image analyses

The rearing temperature had no effect on the forewing or hindwing melanization. Males from both rearing temperatures had significantly lower hindwing black ratios (*p* *<* 0.001) than females. Likewise, both sexes from the H treatment showed significantly less melanized bodies than their L treatment counterparts (*p* *<* 0.005). No significant differences were observed between or within treatments and sexes in the forewing and hindwing areas. Males, however, showed a significantly smaller body area than females (*p* *<* 0.001), as is a typical feature of the species (electronic supplementary material, figure F10S, and tables T18S–T23S).

### (g) Adult condition: heating assay

Our heating assay showed that adults reared in H had a higher heating rate (*p* *=* 0.038), a faster take-off time (*p* *<* 0.005), and a lower body temperature (*p* *=* 0.021) at take-off. Within sexes, both males and females from the H treatment showed faster take-off times (*p* *<* 0.001), and lower final body temperature (*p* *<* 0.005) (electronic supplementary material, figure F10S, and tables T24S–T26S). Hence, the heating assay indicates that adults from H treatment need less time to take-off, can gain temperature faster and achieve flight with lower body temperature than their siblings reared in the L treatment.

### (h) Adult condition: metabolic assay

**Figure 5.**
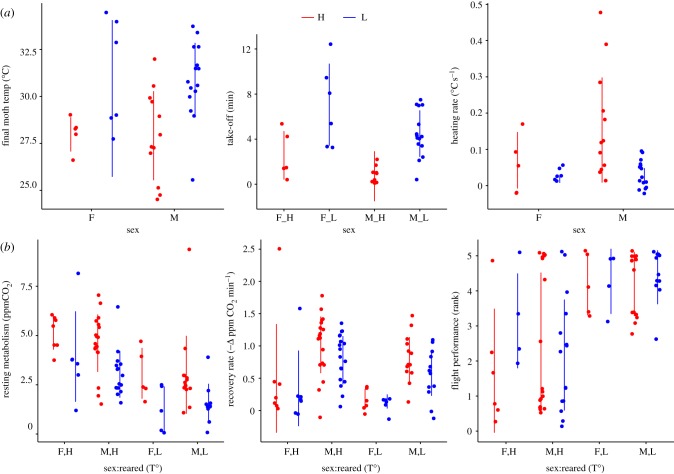
(*a*) Heating assay of *Arctia plantaginis* adults. *x*-axis indicates the sex. (*b*) Flight performance assay of *Arctia plantaginis* adults. *x*-axis shows the sex (M,F) and the temperature treatment (H,L) in which they were reared. Colour indicates the temperature treatment in which the assay was conducted (H, red = 25°C) and low (L, blue = 16°C). (Online version in colour.)

Our results from the metabolism assay showed that both the rearing temperature and the temperature of the assay significantly impacted the resting metabolic rate (*p* *<* 0.001, electronic supplementary material, table T27S). Both females and males reared in the L treatment had lower resting metabolic rate irrespective of whether the assay was conducted in H or L temperatures (figure 5). This suggests that developing in a low temperature can impact the adult's basic metabolic rate. On the other hand, the rearing temperature did not impact significantly on the recovery rate (*p* *=* 0.063, electronic supplementary material, table T28S). However, males from both rearing treatments showed faster recovery rate (i.e. higher -Δ ppmCO^2^ min^−1^) than females, irrespective of whether the assay was conducted in H or L temperature (figure 5; electronic supplementary material, table T28S). Both males and females from the L treatment showed significantly better (*p* *<* 0.001) flight performance while flying in both temperature treatments (figure 5; electronic supplementary material, table T29S).

## 4. Discussion

In this study, we used transcriptomic, phenotypic, metabolic and condition assays to elucidate if responses to environmental conditions experienced by the developing phenotype can be carried over across metamorphosis to the reproductive phenotype. In general, our results partly favour our predicted scenario A, where plastic responses to the thermal environment occur gradually across larval instars, and translate into condition advantages to the adult phenotype. However, not all traits examined were found to be carried over across metamorphosis, and not all that were carried over showed gradual responses during larval development. We did not find the support for the environmental-matching hypothesis.

### (a) Overall gene expression responses

As an ectotherm, the wood tiger moth must be able to respond to environmental temperature variations by adjusting its physiology accordingly. Our transcriptomic analyses showed an impressive capacity for regulation at the gene expression level underpinning such physiology. The overall level of differential gene expression (table 2) and the number of different biological processes being affected in each temperature treatment (electronic supplementary material, figures F6S and F7S), evidence the high level of responsiveness of similar genotypes (i.e. full-sib larvae) to different thermal environments. Other recent gene expression studies looking at developing stages have reported substantially lower differential expression levels than those reported here when considering the magnitude of difference between treatments within each study [6,7,46], However, they have focused only on specific tissues such as silk glands or mid-gut sections, whereas whole larvae were examined here, which might explain the differences.

### (b) Larva–adult melanization

In Lepidoptera, naturally occurring variation in larval melanization has been reported between populations [47], between closely related species [48], and within populations [49]. Melanization plays an important role not only in thermoregulation, but also in immunity (e.g. [50,51]), mate choice [52], mimicry [53,54] and various other physiological functions [53,55–59]. Thus, melanization is a significant contributor to insect fitness. Overall, the larvae gradual darkening in the L treatment (figure 1) was in good agreement with higher expression of melanin synthesis genes in larvae from L, where the candidate genes showed higher expression in samples from L (figure 2). These results highlight a great degree of plasticity in larval melanization, which appears to be a common feature among Lepidopterans. Experimental manipulation has shown that larval pigmentation can be impacted by extrinsic factors such as exposure to UV radiation [14] and hormonal treatments [60] as well as developmental temperature [49,61,62]. In the case of the wood tiger moth, and given our split-family rearing, it can be inferred that larval melanization is not fully genetically controlled, but it can be partly induced by the environment. Such phenotypic responses may allow larvae to cope with rapid variation in Arctic environmental conditions by modifying the amount of black pigmentation across moults, and thereby adjusting its thermoregulation to current local conditions. Previous studies have shown that both in adults and larvae, darker wood tiger moths are more efficient in thermoregulation, but that comes with a cost of less efficient warning signals [63]. More recently, it was found that darker *A. plantaginis* larvae absorb more heat, keep a higher body temperature, and actively avoid overheating by seeking shade sooner than less melanized larvae (M. Nielsen and J. Mappes 2015, unpublished data). These differences in physiology and behaviour could be relevant in the wild where differences in melanization can induce different use and selection of microhabitats.

A high plasticity in melanin deposition during development may also induce trade-offs with other traits because the same genes controlling melanic traits can directly or indirectly affect other traits (i.e. pleiotropic effects) [64]. In our case, a direct effect in melanin change can be foreseen with the warning signal (i.e. the orange patch), whereby decreasing/increasing black pigmentation means the warning signal is more or less visible [65] (figures 1 and 4; electronic supplementary material, figure SF4). The relative costs for larvae to produce the black or the red pigmentation may vary. The red hairs that make up the patch contain eumelanin pigments and traces of diet-derived flavonoids [66]. The black hairs on the other hand, contain only eumelanin [67], which is produced by DOPA initiated by the catalysis of tyrosine [60]. Hence, producing and keeping up the black pigmentation appears costly as resources have to be allocated for its synthesis. Alternatively, the orange hairs may result from a depigmentation or reduced production of eumelanin, allowing flavonoids to be expressed as the main pigment. This could be indicated by the lower expression of melanin genes in larvae after a moult resulting in a reduction of the orange patch size (figure 4; electronic supplementary material, table T13S).

Another indirect effect of plastic melanic pigmentation could be expected with larval immune condition. In Lepidoptera, the link between melanization and immunity is well established, generally showing a positive correlation [68]. Infection experiments in the wood tiger moth have shown than darker larvae are better protected against pathogenic bacteria than less melanic larvae [56]. However, our developmental transcriptome analysis does not fully support this previous result. We found that genes involved in defence responses to bacteria and innate immune response were upregulated during the last instars in the H treatment, when larvae are the least melanic (electronic supplementary material, figure F5S; figure 1). This could reflect an immunity boost as preparation for pupal stage, which could be carried over to the adult stage [69], or a possible mechanism compensating for the scarcity of melanic pigmentation in larvae with large signal sizes. However, this distinction cannot be made in this study as larvae were not directly challenged and tested for immunity responses.

### (c) Life histories

Interestingly, life-history traits impacted during development such as number of moults, time to pupation and time to eclosion did not have an effect on the adult size, and neither the body or wings showed size differences according to rearing temperature (electronic supplementary material, figures F9S, F10S, and tables T14S–T20S). Lepidopterans can show natural variation in the number of larval moults and overall length of their larval phase [70] and challenging environmental conditions often cause an increase of moults (review [71]). Small variation in pupal size but high variation in development time in the wood tiger moth suggests that there might be a threshold weight for pupation as was found in *Manduca sexta* (L.) [72].

During the larval phase, our transcriptome analyses showed processes and molecular functions being impacted differently between the treatments (electronic supplementary material, figures F5S–F7S). Processes such as polarity determination (i.e. orientation of body appendices), cell division and male mating behaviour observed in the H treatment indicate that larvae were in a more advance stage of development than their full-sibs in L (electronic supplementary material, figure F7S). On the other hand, growth-related processes such as chitin binding and structural constituents of the cuticle were found downregulated in the L treatment (electronic supplementary material, figures F5S and F6S) suggesting a slowing down of larval development. However, these regulations did not impact on the size of the adult phenotype (electronic supplementary material, tables T18–T20), indicative of compensatory mechanism during pupation. This is best exemplified by the absence of differentiation in female size between treatments (electronic supplementary material, figure F10S) even though females from L originated from smaller pupae (electronic supplementary material, figure F9S, and table T16S). It is possible that compensatory mechanisms impacted other traits not considered here (i.e. reproductive output) due to rate-limiting resources. It has been shown that smaller wood tiger moth females produce less eggs compared to larger ones [73]. This carried-over effect could have negative consequences to the adult phenotype via an underrepresentation of their genotype in the reproductive cohort and/or if the genotypes have reduced fitness compared to those from larger females.

As with the developing time and adult size, the effect of larval darkening was not found carried over to the adults' wings melanization (figure 1; electronic supplementary material, figure F10S). One explanation could be body segmentation or modularity during development. In insects, adult structures such as wings and legs derive from separate clumps of cells (imaginal disks) that persist through immature development and are only activated via hormones during the pupal stage [74]. Differences in timing of induction of different imaginal disks thus allow independent development of body parts. This could partly explain the uncoupling of larval-wing melanization in our study. Alternatively, different genes may regulate the positioning of melanin pigments in the wings and bodies. Melanization is a two-phase process consisting of pigment biochemical synthesis and its subsequent spatio-temporal positioning. The former can be affected by environmental conditions during development [49,61,62], whereas, in the latter, patterning genes regulate the distribution of pigments [31]. Hence, melanization in larvae and in adults’ wings may occur differently even though melanic elements share the same structural component and develop using the same biosynthesis pathway. A recent study showed that two melanin-related genes can have life stage specific effects, one gene (*chocolate*) affecting only larval melanization, with the other (*melanin*) affecting exclusively adult pigmentation [75]. In addition, genes in the Wnt signalling pathway have been shown to regulate wing pattering in several Lepidoptera species [33,76]. Thus, it can be suggested that in the wood tiger moth, larval and wing melanization are regulated independently, presumably by the activation of different patterning genes or pathways. Moreover, we have previously shown, using neutral genetic markers, that melanization in both fore- and hindwing is temporally stable in both male morphs, and it evolves in a neutral fashion [77]. This indicates that wing melanization could be fixed near its optimum as a component of the adults' warning signal.

The bodies, on the other hand, showed greater melanization in adults reared in L (electronic supplementary material, figure F10S, and table T23S). The role of the body's patterning (i.e. the arrangement of coloured and melanized elements) as a constituent of warning coloration in adult tiger moths has not been investigated. The coloration in the wood tiger moth's bodies is the same as that displayed in their hindwings. Co-variation between colour and melanization may impact perception from a visual predators' point of view, and hence, induce differential selection pressure. In wood tiger moth males, yellow hindwing coloration provides better protection against predators, while white hindwing coloration confers mating advantages [78]. Whether colour polymorphism in bodies has similar or other major effects remains to be investigated. Nonetheless, our results demonstrate that the adult's body melanization can be impacted by the temperature experienced during development.

Although adults with more melanized bodies were produced in the L treatment, this was not translated into condition advantages for adults in the heating assay, in which both males and females from H were able to heat up and take off faster at a lower body temperature (electronic supplementary material, figure F10S, and tables T24S–T26S). This suggests that body melanization may not play a significant role in heat absorbance without light radiation. It has been previously shown that increased melanization in the hindwing, for example, positively affects heating in the wood tiger moth [79]. In this previous study, pinned dead wood tiger moths were exposed to light radiation as a heating source. Here, we examined live individuals and manipulate ambient temperature (electronic supplementary material, figure F2S) to evaluate basal thermal sensitivity. It is unclear if body melanization could have similar heating effects, or if it has more relevance in other functions such as immunity or warning signalling as discussed above. However, our result is illustrative in that ambient temperature experienced during development can impact the adults' ability to engage flight. This indicates that larval and adult basal thermal sensitivities are not decoupled by metamorphosis. In other Holarctic Lepidoptera, *Colias* ssp*.* for example, larvae in different geographical populations and species adapt to local climate via differences in optimal and maximal temperatures for feeding and growth, whereas adults adapt via differences in melanin of the wings [80]. This type of trait decoupling between life stages was not observed in the wood tiger moth, highlighting the importance of developmental temperature for the adults’ flight in this species.

### (d) Metabolism and flight performance

Similarly, the metabolic rate was also impacted by developmental temperature (electronic supplementary material, figure F3S, and tables T27S–T29S). There are two main lines of thought concerning metabolic rate and its interaction with temperature. On the one hand, developing in the cold inevitably depresses rates of biochemical reactions such that cold-adapted genotypes will perform relatively poorly at their low thermal optima (i.e. thermodynamic constraint hypothesis). On the other hand, changes in molecular and cellular structures can compensate for any thermodynamic advantages of high temperatures (i.e. biochemical adaptation hypothesis) and, thereby, cold-adapted genotypes will perform at the same levels as hot-adapted genotypes [81]. We found support for the later hypothesis where adults reared in L outperformed their full-sibs from H in the flight performance assay (figure 5). Moreover, there were no differences in the recovery rate between adults reared in H and L (figure 5, electronic supplementary material, table T28S). This points towards compensatory mechanisms for depressed metabolism during cold rearing. Interestingly, there was no clear differentiation in the expression of flight performance genes between instars of both treatments (figure 3), suggesting that compensatory mechanisms most likely take place during pupation.

Compensatory mechanisms nonetheless could bear costs, especially if compensating for metabolism. Such costs may be represented in the resting or basal metabolic rate, being lower in adults reared in L (figure 5; electronic supplementary material, table T27S). Although the functional significance of a lower resting metabolic rate is unclear, it may correlate with the inferior performance of cold-reared adults in the heating assay where they needed a longer time and higher body temperature to take off (electronic supplementary material, figure F10S, and table T24S–T26S). Other studies have shown that variation in metabolism largely explains flight differences in distance and movement [34,37]. Likewise, higher metabolic rates have been observed in developing larvae due to hot temperatures [82]. Alternatively, or in addition, low resting metabolism could have had an influence on the longer developmental time and a larger number of moults observed in L (electronic supplementary material, figure F9S and table T14S). This could be expected since ontogenetic growth, an energetically costly process, is fuelled by metabolism, and metabolism, in turn, is temperature-dependent in ectotherms.

Our results show that the temperature experienced during development could affect the adult's flight capabilities in different ways. For instance, a higher developing temperature could have benefits on the ability to engage flight as shown in the heating assay where adults reared in H gained temperature faster and were able to take off with lower body temperature than those from L (electronic supplementary material, figure F10S and table T24S–T26S). On the other hand, the ability to sustain flight was superior in adults reared in L, as shown in the metabolic assay where they outperformed the adults reared in H probably because of a lower resting metabolic rate, which potentially allows them to fly in a wider temperature range (figure 5; electronic supplementary material, table T27S.)

## 5. Conclusion

Our integrative study showed a remarkable plasticity of the wood tiger moth at the gene expression, physiological and phenotypic levels. Such plasticity may be part of the reason for its Holarctic-wide distribution. Moreover, our split-family across life stages approach highlights the influence of the environment in partly genetically controlled traits such as melanization. Nonetheless, as developmental resources are limited, plasticity inevitably bears a cost in other traits, in this case, potentially in immunity and warning signal display. In summary, our gene expression and melanization change results in larvae have shown how similar genotypes can respond differently to temperature conditions experienced during development. These different responses do not seem to have an effect on adult traits such as size and wing melanization. However, other adult traits of ecological relevance like heat absorption, body melanization and flight performance were impacted by developing temperature. This indicates only a partial decoupling of pre- and post-metamorphosis life stages, highlighting the importance of evaluating multiple traits across life stages when inferring lifetime fitness. This is especially important for species that exhibit warning colours and experience different selection on body coloration across their different life stages [83].

Future studies should consider full life cycles (i.e. egg-adult), and when possible cross-generational cycles (i.e. egg-adult-egg) since the environment experienced by the adult or eggs could have an impact on the sensitivity to stressors of subsequent life stages. Considering the different responses across life stages can provide a better understanding of ecological and evolutionary adaptations to imminent climate change.

## Supplementary Material

Supplementary Figures

## Supplementary Material

Supplementary tables

## Data accessibility

All sequence data are freely available at the National Center for Biotechnology Information (NCBI, https://www.ncbi.nlm.nih.gov) under BioProject PRJEB14172.
