# Supplementary material for: Evaluating responses to temperature during pre-metamorphosis and carry-over effects at post-metamorphosis in the wood tiger moth (Arctia plantaginis)
Source: Philos Trans R Soc Lond B Biol Sci. 2019 Aug 26;374(1783):20190295. doi: 10.1098/rstb.2019.0295 (PMC6711291; doi:10.1098/rstb.2019.0295)
Supplement: Supplementary Figures [file rstb20190295supp1.pdf]

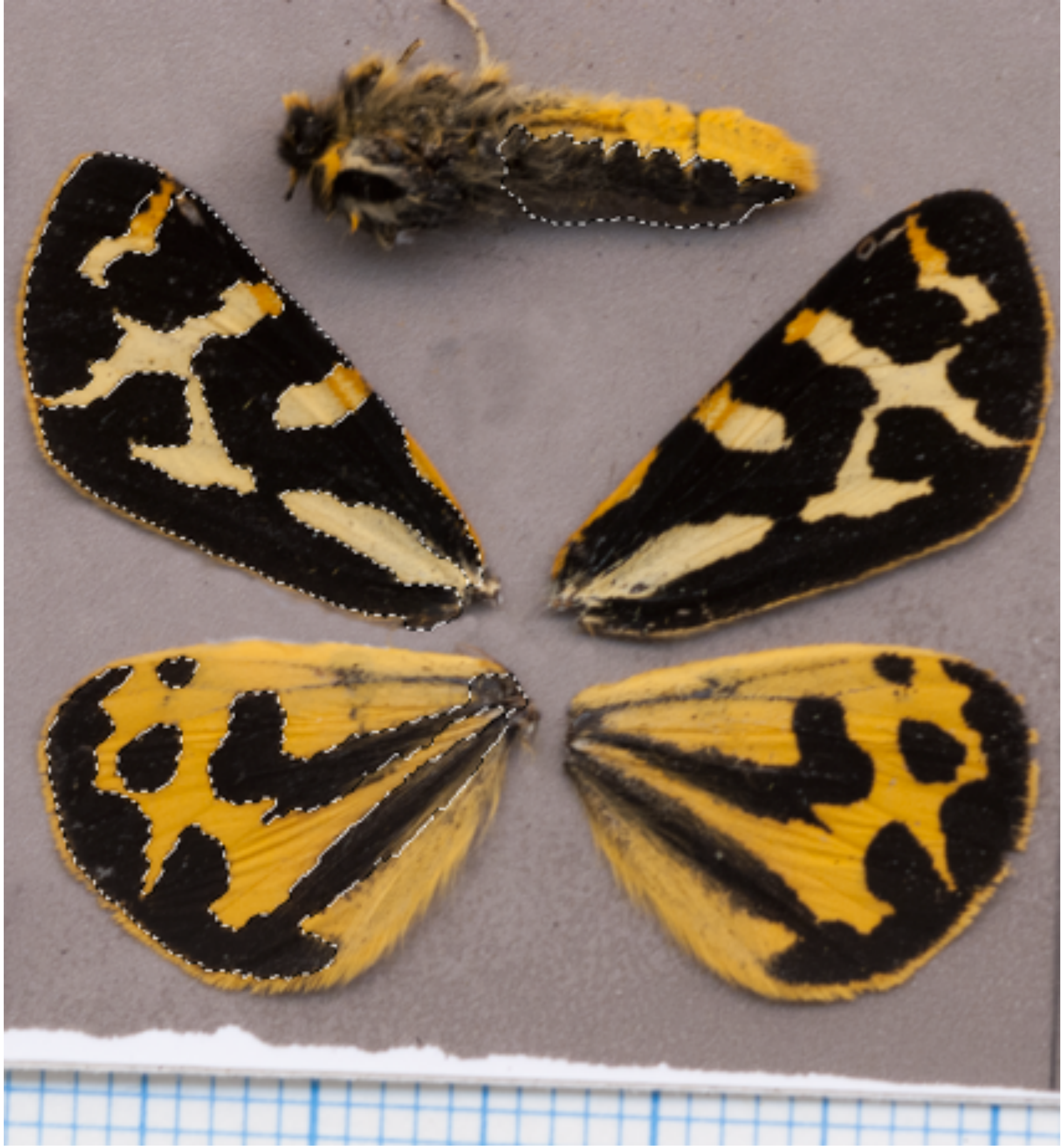

Supporting figure F1S. Selection of melanised areas of wood tiger moth's (*Arctia plantaginis*) wings and body for image analyses.

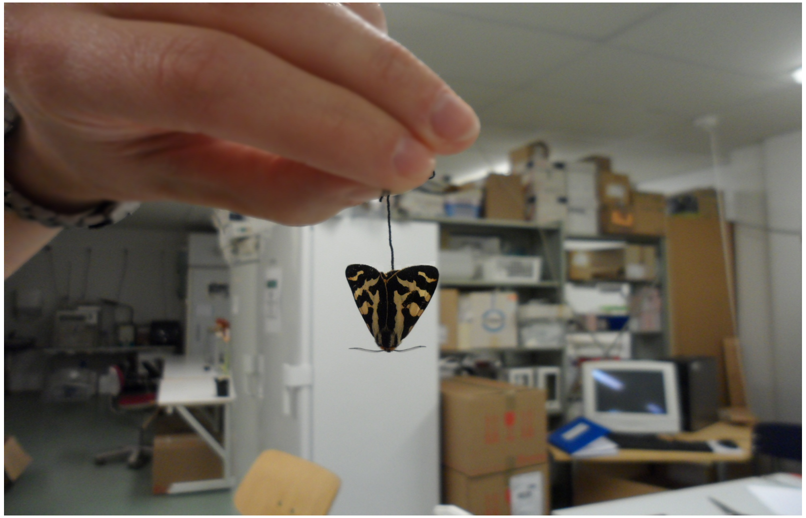

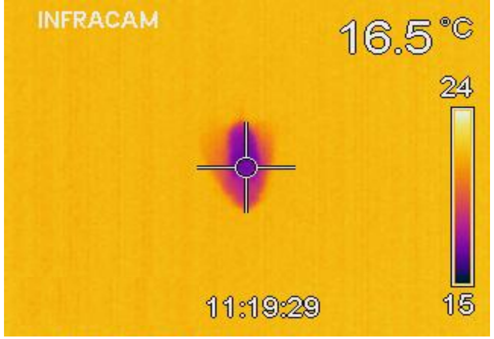

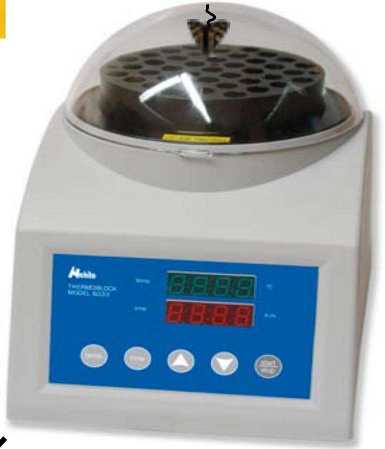

- Attached thread dorsal side
- Measured initial body temp. Infracam
- Place it in thermoblock
- Increase 1C°/min starting at ± 0.5C° of moth's body temperature
- Recorded time to flight
- Measured final body temperature

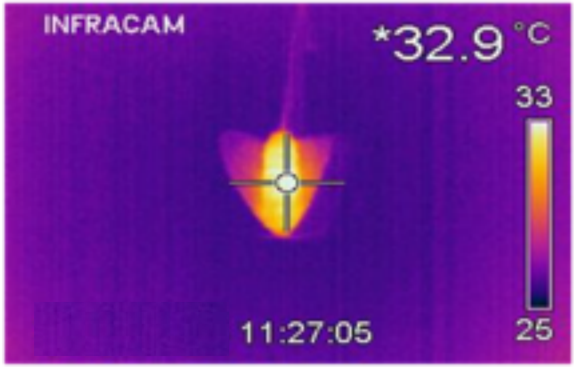

Supporting figure F2S. Heating assay workflow using the wood tiger moth (*Arctia plantaginis*).

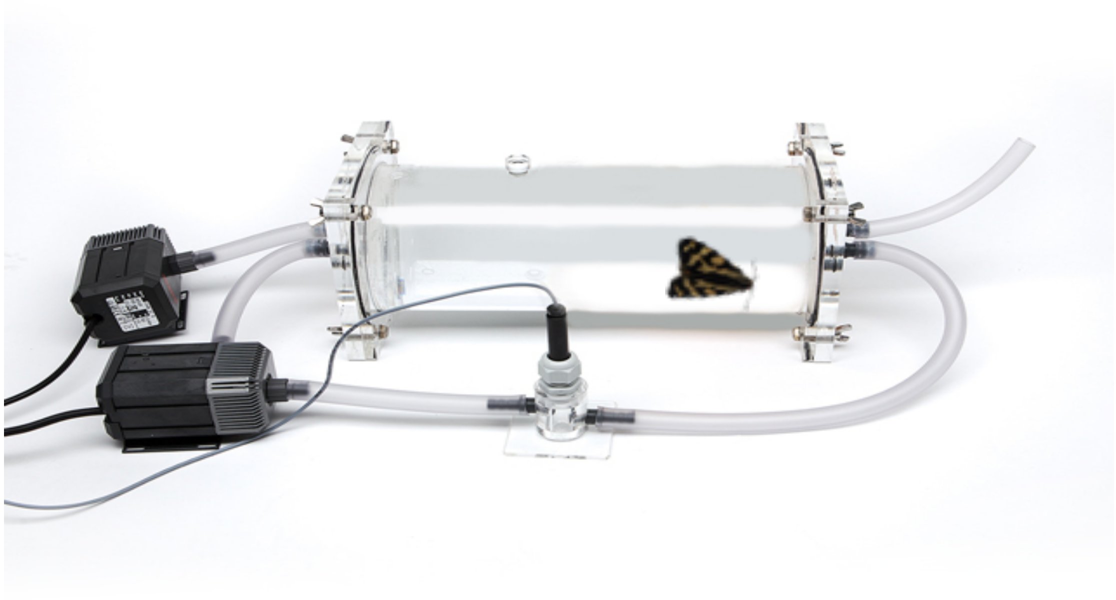

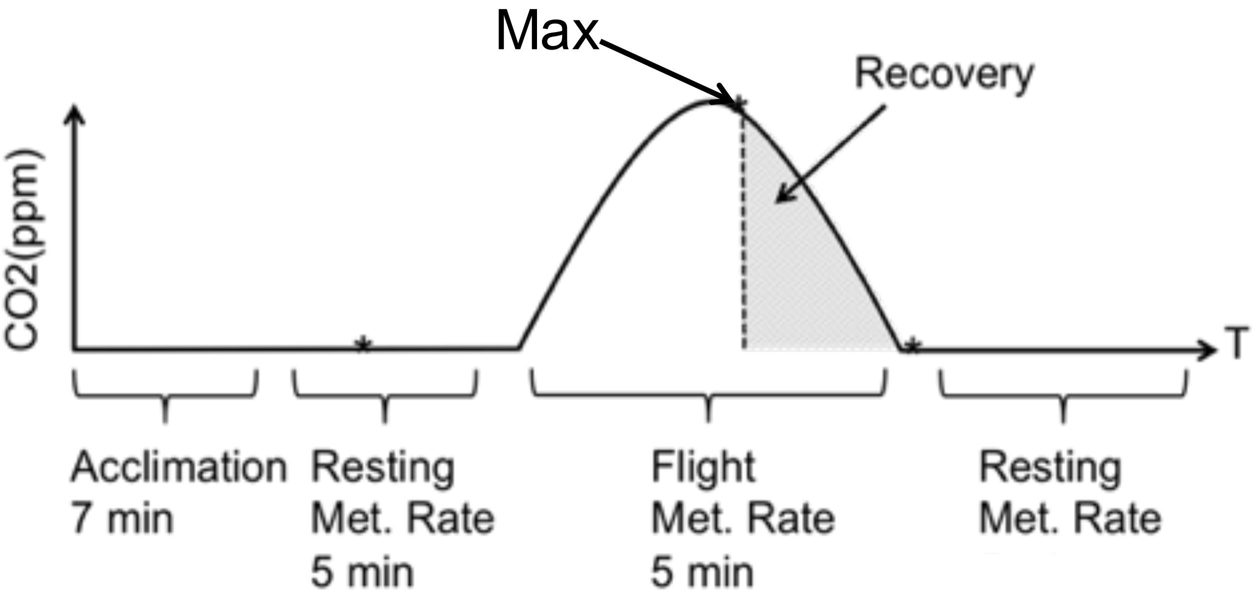

Supporting figure F3S. Metabolic assay workflow using the wood tiger moth (*Arctia plantaginis*).

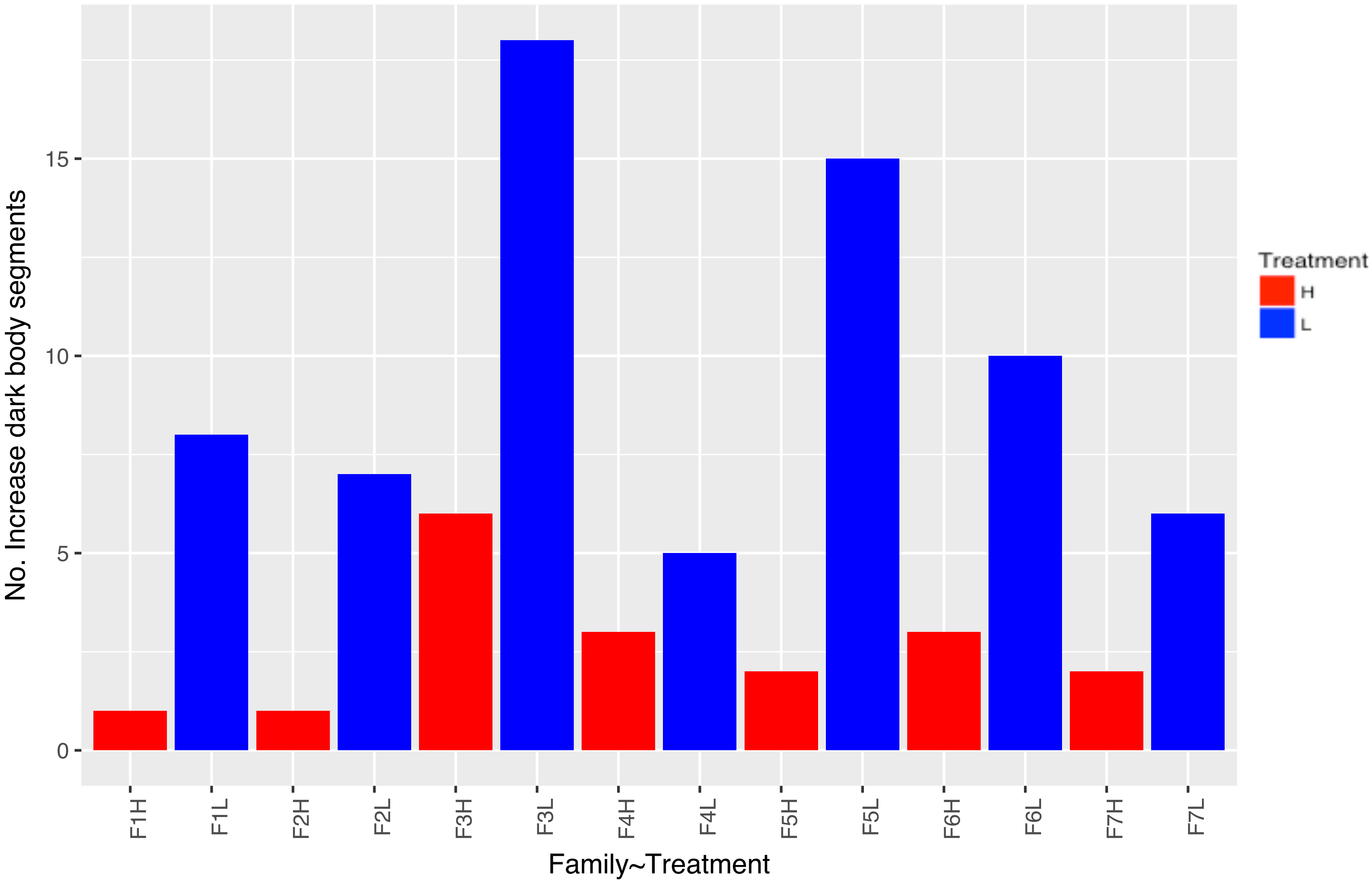

Supporting figure F4S. Number of increases in dark body segments from different families (F) of wood tiger moth (*Arctia plantaginis*) larvae reared in high (H =25C°) and low (T=16C°) temperature.

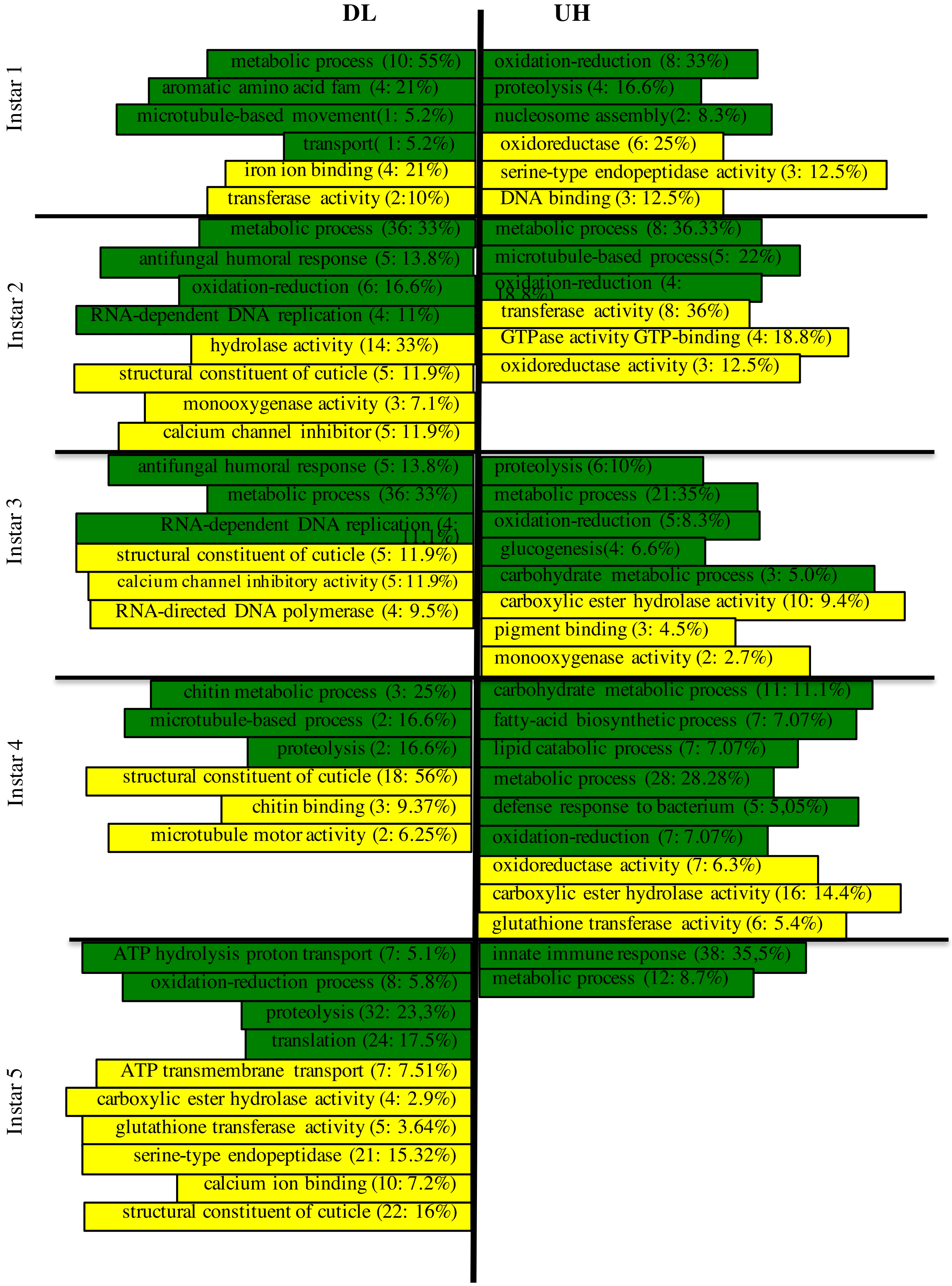

Supporting figure F5S. Biological processes (green) and molecular functions (yellow) of differentially expressed genes in developing instars of *Arctia plantagins* larvae from the high (H) and low (L) temperature treatments. D=down-regulated, U=up-regulated. Numbers in parentheses indicate the number of genes and their percentage contribution to the total number of expressed genes within each instar.

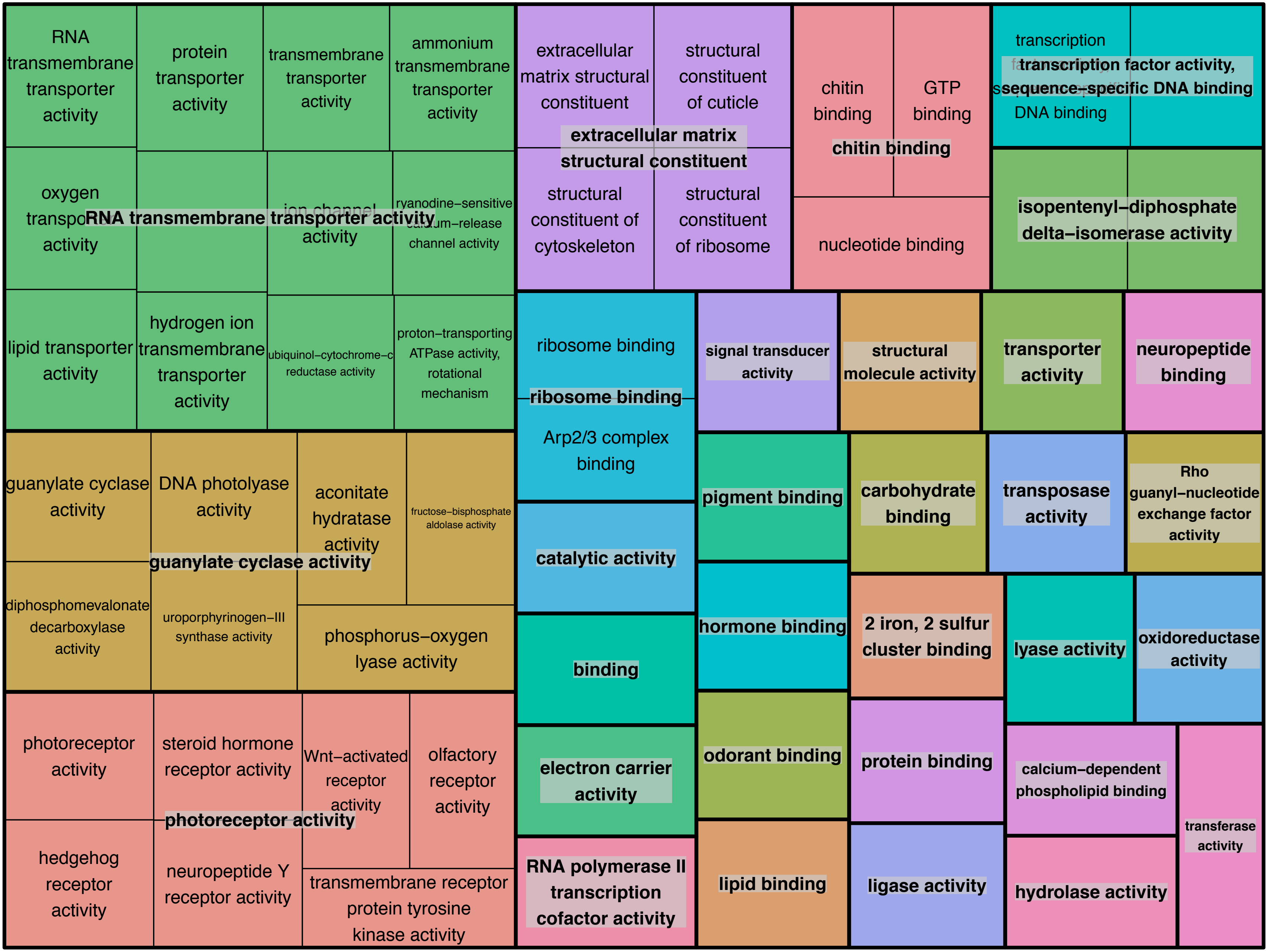

Supporting figure F6S. Biological and metabolic processes of the genes (N=1916) expressed in the low (L) temperature treatment. A colour represents the same process, whereas the size of the square is proportional to the number of genes involved in that process

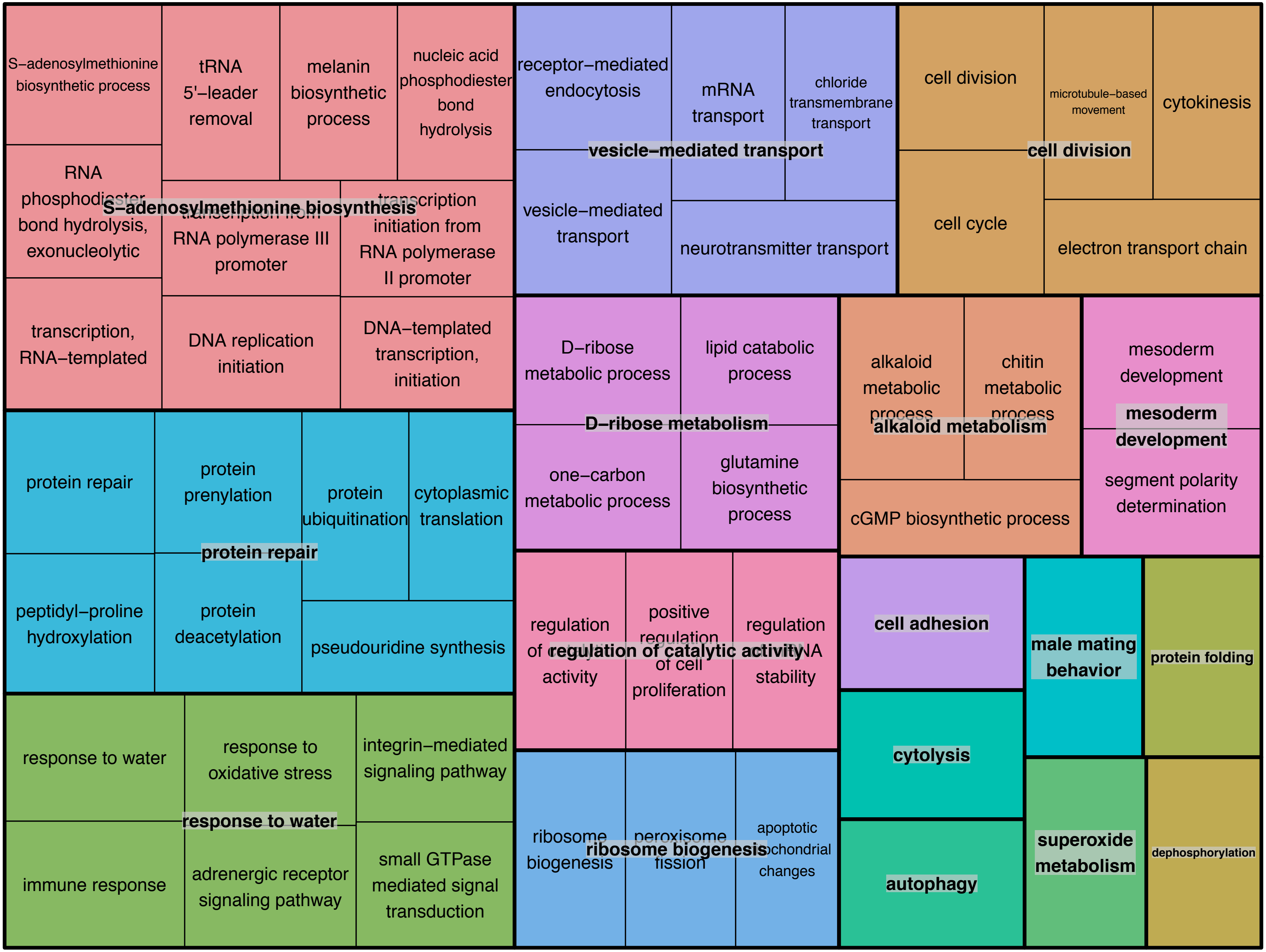

Supporting figure F7S. Biological and metabolic processes of the genes (N=2097) expressed the high (H) temperature treatment. A colour represents the same process, whereas the size of the square is proportional to the number of genes involved in that process

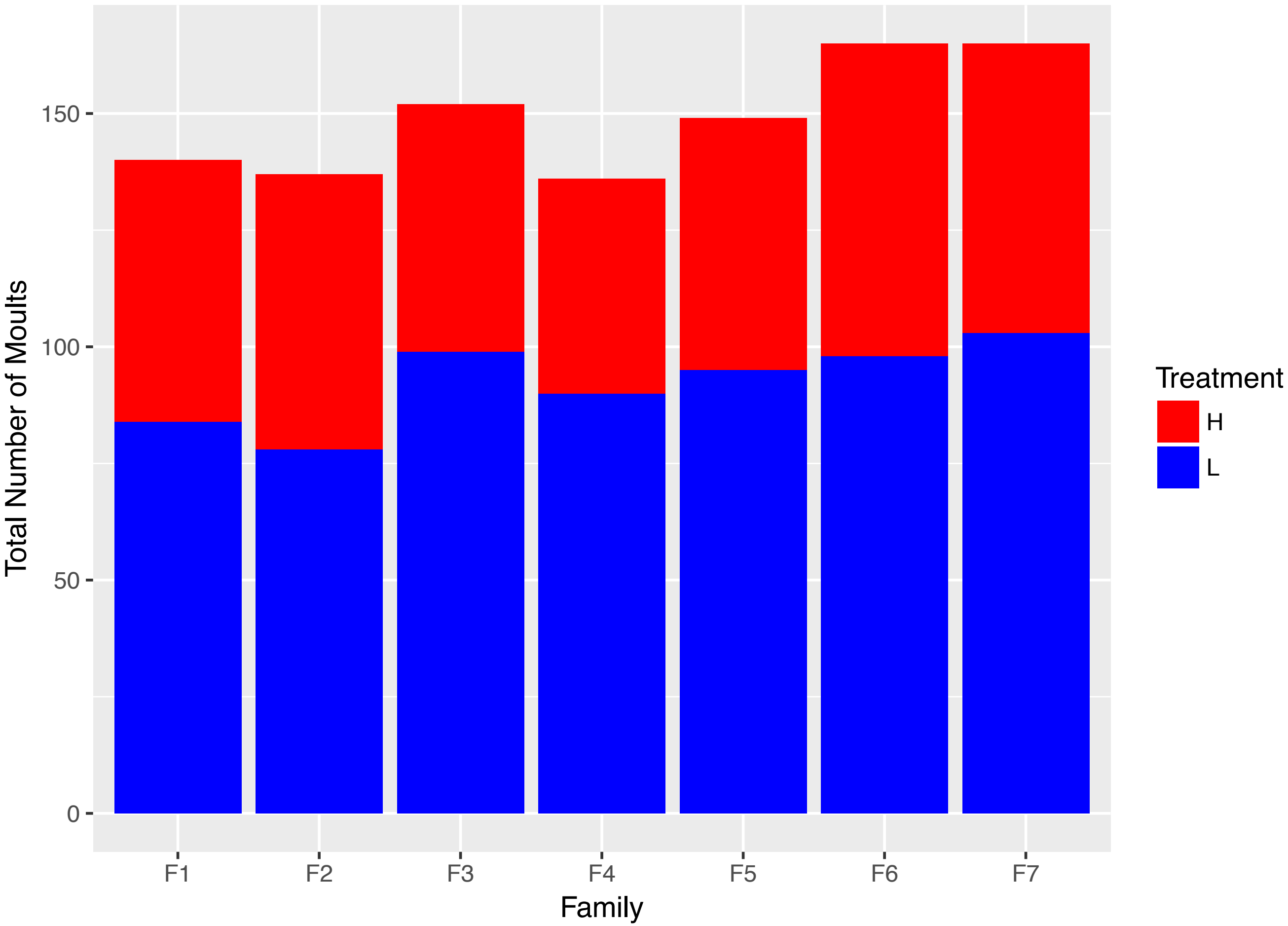

Supporting figure F8S. Total number of moults per family (F) and in (H) and low (L) temperature treatments of wood tiger moth (*Arctia plantaginis*) larvae.

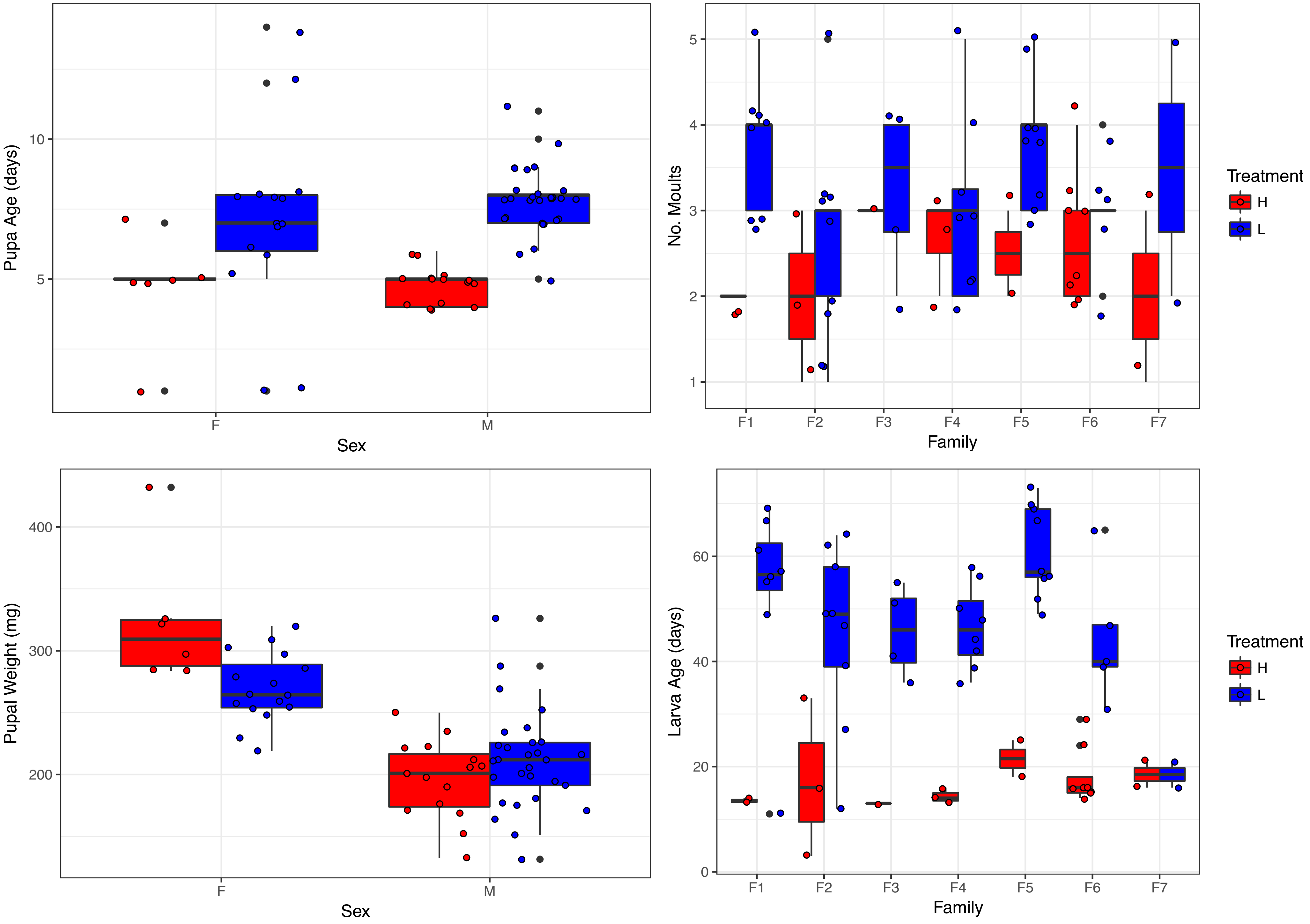

Supporting figure F9S. Life-history traits of *Arctia plantaginis* larvae and pupa within sexes and families reared in high (H=25C°) and low (L=16C°) temperature.

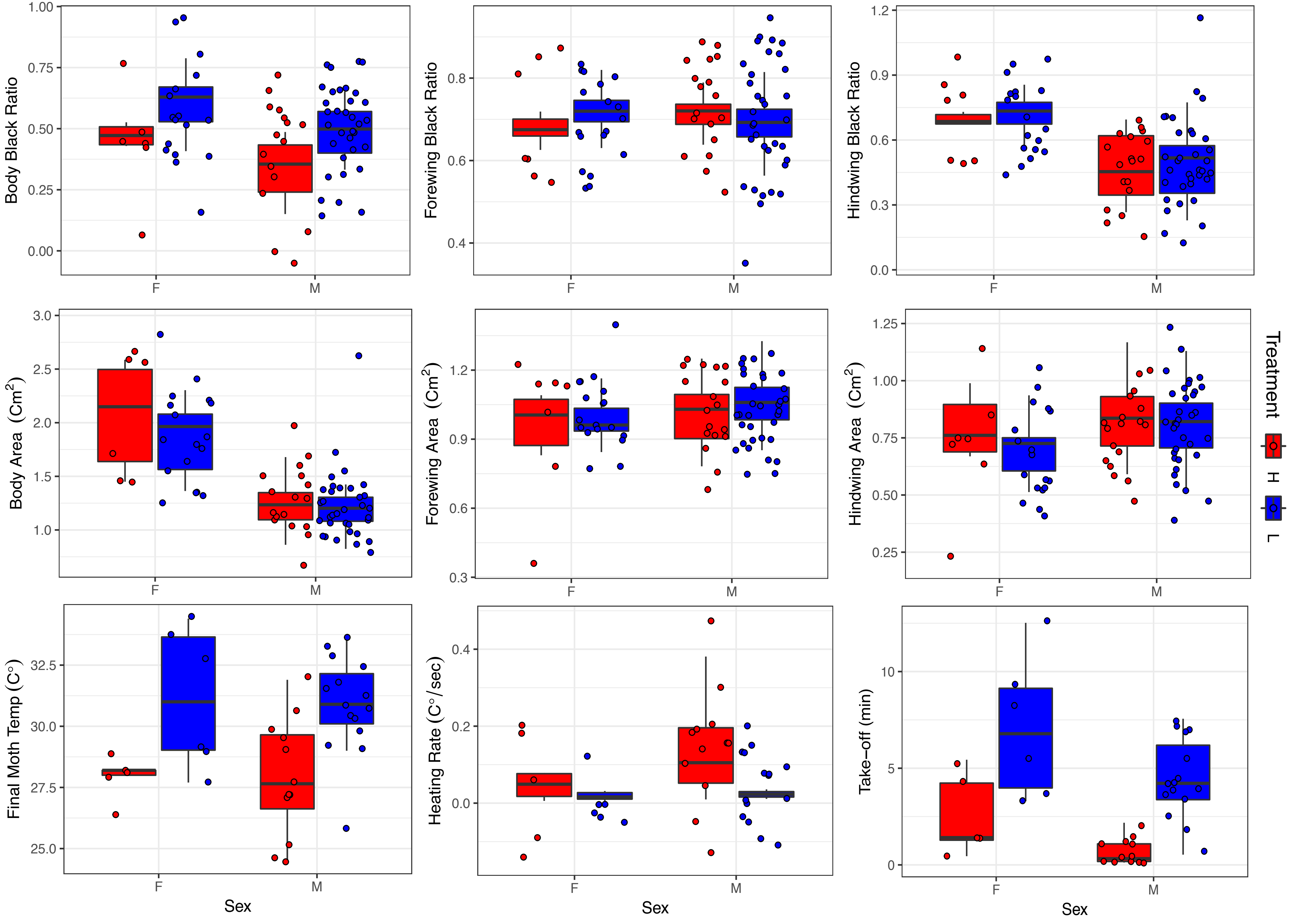

Supporting figure F10S. Body measurements from *Arctia plantaginis* adults reared in in high (H=25C°) and low (L-16C°) temperature.
